# T Cell Receptor Repertoires Acquired *via* Routine Pap Testing May Help Refine Cervical Cancer and Precancer Risk Estimates

**DOI:** 10.3389/fimmu.2021.624230

**Published:** 2021-04-02

**Authors:** Scott Christley, Jared Ostmeyer, Lisa Quirk, Wei Zhang, Bradley Sirak, Anna R. Giuliano, Song Zhang, Nancy Monson, Jasmin Tiro, Elena Lucas, Lindsay G. Cowell

**Affiliations:** ^1^ Department of Population and Data Sciences, UT Southwestern Medical Center, Dallas, TX, United States; ^2^ Department of Neurology and Neurotherapeutics, Department of Immunology, UT Southwestern Medical Center, Dallas, TX, United States; ^3^ Center for Immunization and Infection Research, Moffitt Cancer Center, Tampa, FL, United States; ^4^ Department of Pathology, UT Southwestern Medical Center, Dallas, TX, United States; ^5^ Department of Pathology, Parkland Health and Hospital System, Dallas, TX, United States

**Keywords:** cervical cancer screening, cervical cancer surveillance, immune repertoire, machine learning, regression biomarker

## Abstract

Cervical cancer is the fourth most common cancer and fourth leading cause of cancer death among women worldwide. In low Human Development Index settings, it ranks second. Screening and surveillance involve the cytology-based Papanicolaou (Pap) test and testing for high-risk human papillomavirus (hrHPV). The Pap test has low sensitivity to detect precursor lesions, while a single hrHPV test cannot distinguish a persistent infection from one that the immune system will naturally clear. Furthermore, among women who are hrHPV-positive and progress to high-grade cervical lesions, testing cannot identify the ~20% who would progress to cancer if not treated. Thus, reliable detection and treatment of cancers and precancers requires routine screening followed by frequent surveillance among those with past abnormal or positive results. The consequence is overtreatment, with its associated risks and complications, in screened populations and an increased risk of cancer in under-screened populations. Methods to improve cervical cancer risk assessment, particularly assays to predict regression of precursor lesions or clearance of hrHPV infection, would benefit both populations. Here we show that women who have lower risk results on follow-up testing relative to index testing have evidence of enhanced T cell clonal expansion in the index cervical cytology sample compared to women who persist with higher risk results from index to follow-up. We further show that a machine learning classifier based on the index sample T cells predicts this transition to lower risk with 95% accuracy (19/20) by leave-one-out cross-validation. Using T cell receptor deep sequencing and machine learning, we identified a biophysicochemical motif in the complementarity-determining region 3 of T cell receptor β chains whose presence predicts this transition. While these results must still be tested on an independent cohort in a prospective study, they suggest that this approach could improve cervical cancer screening by helping distinguish women likely to spontaneously regress from those at elevated risk of progression to cancer. The advancement of such a strategy could reduce surveillance frequency and overtreatment in screened populations and improve the delivery of screening to under-screened populations.

## Introduction

The primary cause of cervical cancer is persistent infection with human papillomaviruses (HPV) (1–3). Yet, despite the availability of effective, prophylactic vaccines against HPV (4, 5), cervical cancer remains the fourth most common cancer and fourth leading cause of cancer death among women worldwide (6). In low Human Development Index settings, it ranks second for both measures (6). This is due in part to the fact that current vaccines cover only nine of the most oncogenic HPV types out of over 200 types that have been identified (3). More importantly, however, availability of the vaccine is relatively recent (2006) and is only approved for individuals below the age of 45. Additionally, even within the guideline-approved age groups, vaccination rates remain low. Together, these facts mean that cervical cancer prevalence and mortality will not decrease significantly in the short term (7–9). Thus, general population screening for cervical cancer must continue for the foreseeable future.

General population cervical screening relies on estimating the risk of high-grade lesions or cancer from tests for cervical cell infection by high-risk HPV (hrHPV) types and cervical cytology (Papanicolaou or Pap) tests (10–12). Risk-based management then dictates whether a woman proceeds under general screening, more frequent surveillance screening, or referral for colposcopy, histologic diagnosis, or treatment (11, 12). While these processes have reduced cervical cancer incidence and mortality through the early detection and treatment of cervical precancerous lesions and cancer (10, 13–15), testing improvements providing refined risk estimation would offer significant benefits. The cytology test has low sensitivity to detect precursor lesions, while a single hrHPV test cannot distinguish between a persistent infection and one that the immune system will naturally clear (16). Furthermore, among women who are hrHPV-positive and/or have abnormal cytology, testing cannot identify those who will progress to high-grade cervical lesions or cancer if not treated (17–22). Thus, reliable cervical cancer prevention requires frequent re-testing, referral for colposcopy and possible histologic diagnosis for women in higher risk intervals, and a recommendation for excisional treatment for women with a risk of cervical intraepithelial neoplasia grade 3 (CIN3) of 26% or higher (12). A consequence of this protocol is that women in under-resourced settings or hard-to-reach communities (e.g., immigrant or rural communities) are often under-screened and therefore at risk of undetected precancerous lesions and cancer (23–26). In contrast, in adequately screened populations, there is a potential for overtreatment, with its associated risks and complications (27–31). Methods to improve risk assessment for cervical cancer, particularly assays that can predict regression of precursor lesions or clearance of HPV infection, would be expected to benefit both populations. Improving the effectiveness of less-frequent testing could enable improved strategies for delivering screening to under-screened populations, thereby reducing cervical cancer incidence and mortality (23–26). In screened populations, improved risk assessment could reduce overtreatment.

We hypothesized that profiling adaptive immune responses, particularly T cell responses, could provide an additional axis along which to assess CIN3 and cancer risk due to the role of these responses in clearing HPV infections (32, 33) and resolving both low- and high-grade lesions (34–38). Indeed, such T cell responses are also associated with positive outcome even after cervical cancer has developed (37, 39–43). In order to profile these responses, we selected high-throughput adaptive immune receptor repertoire sequencing, because it has become one of the primary techniques for profiling adaptive immune responses in cancer (44) and for identifying potential immune biomarkers (45–50).

The goal of this study was two-fold. First, we aimed to determine whether T cell receptor (TCR) repertoire profiling could be conducted on cervical cytology samples, the sample type routinely collected for cervical cancer screening and surveillance and on which cytology tests and hrHPV tests are conducted. The ability to conduct TCR repertoire profiling on this sample type would greatly facilitate incorporation of TCR profiling into current screening protocols. Second, we aimed to determine whether predictive signatures could be identified in these repertoires, signatures that are predictive of a patient’s cytology/hrHPV test results at their next round of testing. We are aware of only two papers that have described TCR repertoire profiling in the context of cervical lesions or cancer, and both differ from the current study in important ways. Lang Kuhs et al. (51) compared peripheral blood TCR repertoires between women who had cleared an HPV-16 infection and women who had HPV-16-related CIN3 or higher (including invasive cancer cases). Cui et al. (52) compared the peripheral blood repertoires between three groups of women: women with cervical cancer, women with CIN of any grade, and healthy women. Cui et al. (52) additionally sequenced tumor and lymph node repertoires from women with cervical cancer. Both studies compared repertoire-level summary statistics, such as diversity and gene segment usage. Cui et al. (52) additionally examined repertoire overlap between samples and between groups. While TCR repertoire sequencing from blood, tumor, and lymph node samples is routine, we are the first to demonstrate successful TCR sequencing from cervical cytology samples. This is important not just because such samples are already routinely collected for cervical screening and surveillance, but also because the cervical repertoire is expected to be more representative of the T cells acting in the local immune response against HPV infection and abnormal lesion cells (53). Furthermore, while both studies (51, 52) make the important contribution of demonstrating differences in peripheral blood T cell repertoires between stages along the cervical cancer spectrum, these differences are reflective of different phenotypes possessed by the donors at the time of sample collection and are not predictive of the patients’ disease trajectories.

**Figure 1:**
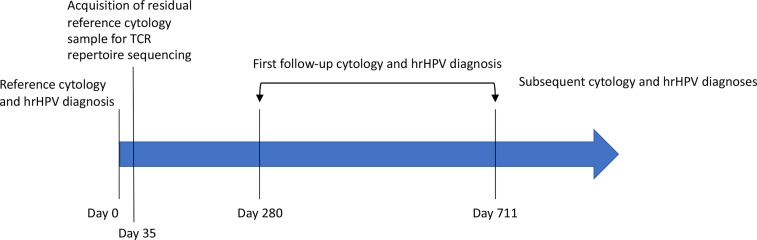
The timeline of prospective follow-up diagnosis collection. Residual, standard-of-care cytology samples were rescued at the time of scheduled discard, five weeks (35 days) after their collection and the completion of their use for clinical decision making. The associated cytology and hrHPV diagnoses constitute the patient’s reference diagnosis (**Table 1**). These samples were used for HPV genotyping and TCR repertoire sequencing. The electronic health record was periodically checked for cytology, HPV, and pathology reports related to cervical cancer screening. Twenty-four women returned for follow-up screening 280-711 days after collection of the index sample (**Table 1**). Seven of those women have returned for a second follow-up screening (**Table 1**). The remaining women have not returned.

In this proof-of-principle study, we demonstrate the ability to obtain sufficient DNA for TCR repertoire profiling from cervical cytology samples collected during general and surveillance screening. Additionally, a subset of the women have had a second cervical sample collected 280-711 days after the one on which we conducted sequencing (**Figure 1**), allowing us to determine whether they experienced a transition to cytology/hrHPV test results expected to have a lower CIN3 or cancer risk compared to the index results. Indeed, we found that women with lower risk at follow up had evidence of more robust T cell clonal expansion in the index sample than women with persistent risk. Furthermore, applying a machine learning-based method we previously developed (45–47, 50), we identified a biophysicochemical motif in the complementarity-determining region 3 (CDR3) of TCR β chains (TCRB) whose presence was associated with lower-risk follow-up test results with 95% accuracy by leave-one-out cross-validation (19 of 20 trajectories correctly predicted). These results suggest that sequencing TCR repertoires from the cervical samples used for cytology and hrHPV testing could improve cervical cancer screening by further refining the risk estimates used for personalized risk-based management. Furthermore, these results represent a significant advance in the field of immune repertoire predictive modeling as the first to demonstrate the potential to predict a clinically relevant future outcome.

## Materials and Methods

### Sample Collection

All samples for this study were obtained according to protocols approved by the Institutional Review Board of the University of Texas Southwestern Medical Center. Cervical samples were obtained during routine general and surveillance cervical cancer screening at Parkland Health and Hospital System (PHHS). Samples were collected *via* the ThinPrep technique using a spatula or brush and PreservCyt solution. Samples were processed for clinical decision making and held for five weeks, at which point they are typically discarded. We obtained the residual samples at the time of their scheduled discard (**Figure 1**).

We included samples from White Hispanic women age 18 years or older. Given the racial/ethnic disparities in cervical cancer incidence and mortality, we decided to eliminate this confounding factor in this proof-of-principle study by focusing on a single race/ethnicity and selected White Hispanics as the focus group, because approximately 70% of the patients who seek care at PHHS are White Hispanic. We excluded women who were HIV+, pregnant, had an intrauterine device, or had a sexually transmitted disease at the time of sample collection. We obtained samples across all cytology result categories (10, 54, 55): Negative for Intraepithelial Lesion or Malignancy (NILM, Normal), Abnormal Squamous Cells of Undetermined Significance (ASCUS), Low-grade Squamous Intraepithelial Lesion (LSIL), and High-grade Squamous Intraepithelial Lesion (HSIL). At PHHS, the primary screening strategy is cytology alone with a reflex hrHPV test for women with an ASCUS cytology result. The test assays for positivity across 14 HPV types, and the ASCUS result category is divided into ASCUS/HPV- (negative for all 14 types) and ASCUS/HPV+ (positive for at least one type). An additional exclusion criterion was applied to women with a result of Normal, ASCUS/HPV-, and ASCUS/HPV+, and that is they were excluded if they had previously had cervical cancer or previous treatment of cervical pre-cancerous lesions.

We applied these inclusion and exclusion criteria in a quota sampling scheme to ensure adequate representation of women across all five result categories. We targeted a minimum of 100 samples total with a minimum of 15 samples in each category, and then rescued all samples meeting our criteria each week until all minimums were reached.

### DNA Isolation, TCRB Sequencing, and HPV Genotyping

Genomic DNA was isolated using the DNeasy Blood & Tissue kit (Qiagen) following the manufacturer’s guidelines. Briefly, cervical samples in PreservCyt solution were spun down and dissolved in ATL buffer with proteinase K at 56°C. After addition of AL buffer and 100% ethanol, the mixture was passed through the DNeasy Mini spin column. After two rounds of washing, DNA was eluted in ultrapure water. DNA concentration and integrity were determined by a NanoDrop One Microvolume UV-Vis spectrophotometer (Thermo Fisher Scientific).

For each sample, two micrograms of DNA were sent to Adaptive Biotechnologies (AB) for TCRB sequencing at survey depth. Pre-processing, inference of germline gene segments, determination of CDR3 regions, and whether rearrangements are productive or non-productive was conducted by AB. Results were made available in a tab-separated value file which we downloaded for continued analysis. Further analysis was conducted on this downloaded data using VDJServer (http://vdjserver.org) (56) and GraphPad Prism version 8.4.2, as specified below.

One hundred nanograms of DNA were sent to the Center for Immunization and Infection Research at Moffitt Cancer Center for HPV genotyping. The samples were tested per protocol using the Roche Linear Array HPV genotyping test (Roche Diagnostics) for the presence of HPV as described in (57–60). Briefly, samples were amplified using an Applied Biosystems Veriti 96 well thermal cycler according to the instructions of the protocol, and all samples were genotyped regardless of the accompanying HPV PCR gel result. This assay uses an HPV L1 consensus PCR primer to detect 37 HPV genotypes, and the strips were interpreted using the kit-included overlay. The genotypes that can be detected with this assay are: 6, 11, 16 18, 26, 31, 33, 35, 39, 40, 42, 45, 51, 52, 53, 54, 55, 56, 58, 59, 61, 62, 64, 66, 67, 68, 69, 70, 71, 72, 73 (MM9), 81, 82 (MM4), 83 (MM7), 84 (MM8), IS39, and CP6108.

### Statistics

Statistical analyses were conducted in GraphPad Prism version 8.4.2 for macOS. The Kruskal-Wallis test was used to test for differences between group medians for tests with more than two groups (i.e., the five result categories). In the case of a statistically significant result with the Kruskal-Wallis test, Dunn’s *post hoc* multiple comparison test was used to identify the specific pairs of groups with different medians. Multiple comparison-adjusted p values are reported. For tests with two groups, the Mann Whitney test was applied. To test for equality of variances, we utilized the Brown-Forsythe test by calculating the absolute deviation between each value and its group median and then conducting a one-way Analysis of Variance (ANOVA) on the absolute deviations. In the case of a statistically significant result with the one-way ANOVA, Tukey’s *post hoc* multiple comparison test was used to identify the specific pairs of groups with different means. Multiple comparison-adjusted p values are reported.

### Statistical Classifier

To identify TCRB sequence patterns that distinguish cervical TCR repertoires collected from women with an abnormal cytology/hrHPV result who either did or did not transition to a lower risk result on a subsequent test, we leveraged an approach we previously developed (45–47, 50). The approach was developed to identify sequence patterns that distinguish (1) B cell receptor repertoires from patients with Multiple Sclerosis versus those from patients with another neuroinflammatory disease (45), (2) TCR repertoires collected from breast or colorectal cancer TILs versus those from adjacent healthy tissue (46), and (3) TCR repertoires collected from high-grade serous ovarian cancer tissue versus those from healthy ovarian tissue collected from cancer-free women (50). Briefly, as described and motivated in (46), we focused on the CDR3 region of each TCRB sequence, excluded the first and last three residues of each CDR3, and represented the remaining sequence as the set of every four contiguous amino acids (4-mer) it is composed of. To enable identification of 4-mers with different amino acid sequences but capable of binding the same set of peptides, we represented each 4-mer using numerical values for the biophysicochemical properties of its component amino acids. We used Atchley factors as the biophysicochemical descriptors, which correspond to polarity, secondary structure, molecular volume, codon diversity, and electrostatic charge (61).

**Figure 2:**
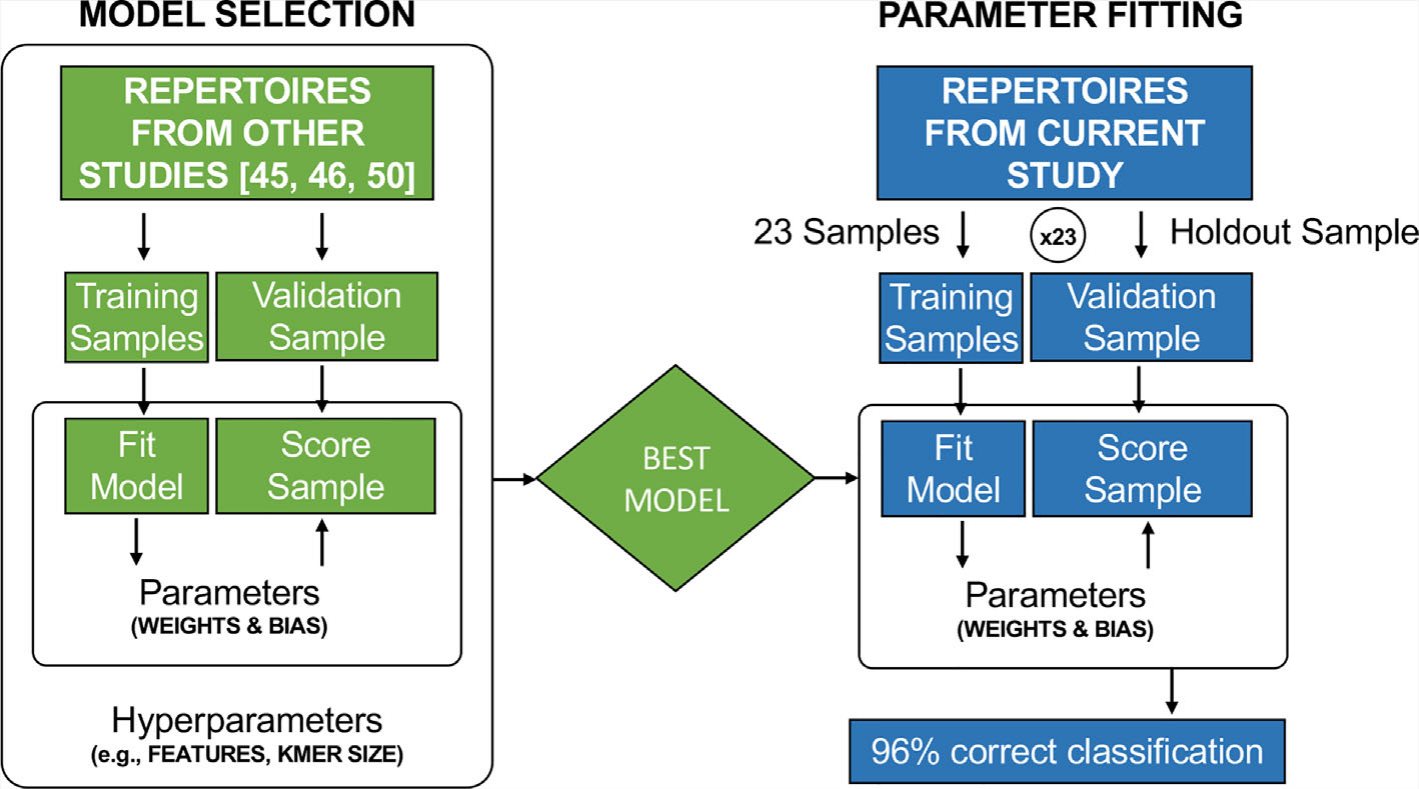
Model selection was conducted in earlier studies (45, 46, 50) to determine the best representation of TCR CDR3 sequences, such as the best k-mer size and the use of Atchley Factors rather than a DNA or AA sequence representation. The cervical cytology samples used in this study were not included in the model selection process. In this study, the previously identified best model was used, and the weights and bias term were fit by gradient descent in leave-on-out cross-validation.

As in (46, 50), each amino acid in a 4-mer was represented by a vector of its five Atchley factor values, and we included an estimator of 4-mer relative abundance. This resulted in a numerical vector representation of length 21 for each 4-mer. As in (50), each element of the vector was normalized to zero mean and unit variance. We then used a logistic model to calculate the probability that a 4-mer derives from a sample whose donor transitioned to lower risk results. To estimate the probability that the entire sample derived from a donor who transitioned to lower risk results, we took the maximum probability calculated over all 4-mers in the sample, based on Multiple Instance Learning (45, 46, 50, 62). In both cases, a probability greater than 0.5 results in a model-assigned label of transitioning to lower risk. Thus, any 4-mer with a probability > 0.5 is assigned this label, and any sample with at least one 4-mer with a probability > 0.5 is assigned this label. Samples labeled as not transitioning to lower risk do not have a single 4-mer with probability > 0.5. Weights on each of the 21 4-mer features along with a bias term were fit by gradient optimization (**Figure 2**). While a probability threshold of 0.5 was set for classification, the inclusion of a bias term results in the effective threshold being set as part of parameter fitting. As in (50), initial values for the weights were drawn from a normal distribution and the bias was initialized to zero. We ran gradient optimization for 2,500 steps to maximize the likelihood that each model-assigned label was correct. Optimization was run from 524,288 different initial values. The best set of weight values was determined by exhaustive leave-one-out cross-validation where one sample (i.e., all of its sequences) was left out each round (**Figure 2**). Early stopping was used to reduce overfitting, as this regularization approach performs best for this model (46). However, the best performance was observed at the last iteration. We note that this process was used to fit parameter values. There was no model selection; only a single model was considered (**Figure 2**).

The model’s accuracy was assessed by its performance on the held-out samples as described above. To examine the motif properties that result in a model-assigned label of transitioning to lower risk, (i.e., the model predicts that the woman from whom the index sample was collected will have lower-risk results on the first follow-up test), we retrained the model on all 24 samples.

## Results

### Patient and Sample Characteristics

**Figure 3:**
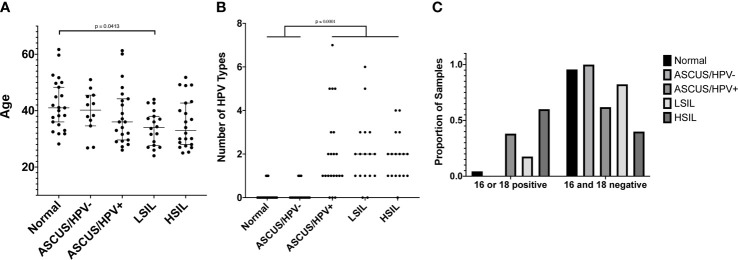
**(A)** The age of women at the time the cervical sample was collected. Age was only available for 97 of the 103 women whose cervical sample was used for TCRB sequencing. Age is shown on the y-axis, and the result category, according to the cervical cytology and hrHPV test, is shown on the x-axis. A test for difference in the median value between result categories was conducted using a Kruskal-Wallis (KW) test followed by a Dunn’s *post hoc* multiple comparison test if the KW p value was ≤ 0.05. The Dunn’s multiple comparison-adjusted p values are reported when p ≤ 0.05. **(B)** The number of HPV types for which a sample tested positive using the Roche Linear Array. Only 96 of the 103 samples whose cervical sample was used for TCRB sequencing had sufficient DNA to also conduct HPV typing. The number of HPV types for which a sample tested positive is shown on the y-axis, and the result category, as defined in **(A)**, is shown on the x-axis. Statistical tests were conducted and reported as in **(A)**. **(C)** The proportion of samples in each result category, as defined in **(A)** that are positive for HPV16, HPV18, or both (left grouping) or are negative for both (right grouping), according to the Roche Linear Array. The proportion of samples is shown on the y-axis and indicated by the bar height. The result category is indicated by the bar color and legend. A Chi-square test of independence resulted in p < 0.0001.

We obtained a total of 103 de-identified samples meeting our inclusion/exclusion criteria and on which TCRB sequencing was conducted. This included 23 Normal, 18 ASCUS/HPV-, 22 ASCUS/HPV+, 18 LSIL, and 22 HSIL samples. While the age range was similar across the categories, there was a slight trend toward lower ages with higher risk result categories (p = 0.0282 by Kruskal-Wallis test, **Figure 3A**). Dunn’s post-hoc multiple-comparison test revealed a single significant pairwise difference between the Normal and LSIL categories with median ages of 41 years and 34 years, respectively (p = 0.0413). Note age was only available for 97 of the 103 women whose sample was used for TCRB sequencing (**Supplementary Table 1**).

The protocol for HPV testing at PHHS is to test only those samples with an ASCUS result. The test includes only 14 types, and the reported result is only one of negative for all types or positive for one or more types. Thus, we elected to have more comprehensive HPV genotyping conducted for the 96 of the 103 samples that had sufficient DNA for both HPV genotyping and TCRB sequencing (**Supplementary Table 1**). Unsurprisingly, we found that the vast majority of Normal samples (20 of 23) tested negative for all 37 types included in the Roche Linear Array (**Figure 3B**). We further found that the median number of types with which an individual was infected was higher in the more severe result categories, with individuals in the Normal (median of 0 types) and ASCUS/HPV- (0 types) categories being infected with significantly fewer types than those in the ASCUS/HPV+ (1 type), LSIL (2 types), and HSIL (2 types) categories (p < 0.0001 by Kruskal-Wallis test, p ≤ 0.0001 by Dunn’s post-hoc multiple-comparison test, **Figure 3B**). Additionally, we found that the proportion of samples positive for HPV16, HPV18, or both was significantly higher for the ASCUS/HPV+ and HSIL categories than the other categories (p < 0.0001 by Chi-square test, **Figure 3C**).

### TCRB Repertoire Properties Do Not Differ by Result Category or HPV Positivity

We sent all 103 samples to AB for TCRB sequencing and obtained robust template counts for the majority of samples, where a single template corresponds to a single rearranged TCR gene before PCR amplification. The total number of productive templates, i.e., templates expected to encode a functional TCRB protein, was 284,663, for an average of 2,764 per sample. There was, however, considerable sample-to-sample variability (**Supplementary Figure 1A**). Thirty-eight samples had productive template counts below 1000; 21 of these had productive template counts below 500. Fifteen had productive template counts above 5000. The counts and variability are consistent with what we have observed with other clinical samples from non-blood and non-lymphoid tissue samples.

AB provides the following measures that are expected to correlate with lymphocyte abundance in the cervical sample (63–65) and, by extension, at the site of cervical sample collection: Productive Templates, as defined above; Productive Rearrangements, defined as a unique nucleotide sequence generated through V(D)J recombination and representative of a unique clonal lineage; and Fraction Productive of Cells Mass Estimate, defined as the estimated fraction of nucleated cells in the sample that are T cells. Subsequently, these measures are referred to as templates, rearrangements, and the estimated T cell fraction. For each of these measures, we found no difference in either the median value or variability about the median between the five result categories (**Supplementary Figure 1**). Additionally, when we pooled all HPV+ samples, regardless of type, and all HPV- samples, we found no difference in the median values between the HPV+ and HPV- groups for any of these measures (**Supplementary Figure 2**). Statistical tests were performed with and without the samples with template counts below 500, and the results were the same.

When T cells encounter antigens for which their TCRs have specificity, they respond by proliferating to form a clone of cells expressing identical TCRs. This is referred to as clonal expansion. AB provides measures expected to be associated with the extent of clonal expansion. These measures are: Percent Unique Productive, defined as the percentage of productive templates that are unique (# Productive Rearrangements/# Productive Templates); Max Productive Frequency, defined as the relative abundance of the largest clone; and Normalized Productive Clonality, defined as 1 – normalized Shannon Entropy and which captures the extent to which the distribution of all relative clonal abundances has shifted away from a uniform distribution as a consequence of clonal expansion. Subsequently, these measures are referred to as the percent unique, the largest clone size, and clonality. For each of these measures, we found no difference in either the median value or variability about the median between the five result categories (**Supplementary Figure 3**). As above, when we pooled all HPV+ samples, regardless of type, and all HPV- samples, we found no difference in the median values between the HPV+ and HPV- groups for any of these measures (**Supplementary Figure 4**). Statistical tests were performed with and without the samples with template counts below 500, and the results were the same.

### The Size of the Largest T Cell Clone Differs Between Women Who Do and Do Not Transition to Lower-Risk Results on Follow-Up

After demonstrating that robust TCR sequencing data could be obtained from cervical cytology samples, we sought to determine whether TCR repertoire properties from these samples (index samples) were associated with follow-up cytology/hrHPV results obtained after sample collection and sequencing (**Figure 1**). Thus, we obtained all available follow-up cytology and reflex hrHPV test results for all 103 women and classified the women into two groups on the basis of whether the first follow-up results showed evidence of transitioning to a lower risk category relative to the index results. Twenty-four women had a second cytology sample collected 280-711 days after the one on which we conducted sequencing (**Table 1**). Among them, 18 experienced a transition to a lower risk category. Two transitioned from an HSIL to an ASCUS cytology result, and the remainder transitioned to normal cytology (**Table 1**). Both of those who were ASCUS on first follow-up were normal on second follow-up (**Table 1**). Thus, all 18 ultimately transitioned to normal cytology. Fourteen of 18 had a reflex hrHPV result on first follow-up, nine of which were hrHPV- (**Table 1**). Two of those that were positive on first follow-up were negative on second follow-up (**Table 1**). Thus, 11 of 14 were ultimately hrHPV-. Four had no follow-up reflex hrHPV test during the period of this study. Of the six we classified as having persistent risk relative to the index results, all six were LSIL or HSIL on first follow-up (**Table 1**). All four with LSIL cytology had a reflex hrHPV test, and three of them were positive (**Table 1**).

**Table 1:** The first (column 3) and second (column 7) cytology and hrHPV test results for the 24 women who had two sets of results.

Sample ID	Age	Reference Diagnosis	HPV Type	Treatment	Time to Follow-up Diagnosis (days)	Follow-up Diagnosis	Assigned Cancer Risk Label	Model Probability of Reduced Risk	Predicted Cancer Risk Labe	Diagnoses After the First Follow-up
2_30	43	ASCUS/HPV-	Negative		710	Normal/HPV-	Reduced	0.69	Reduced	
2_31	51	ASCUS/HPV-	Negative		376	Normal	Reduced	0.69	Reduced	
3_06	44	ASCUS/HPV+	18,39,53		428	*Normal/HPV+	Reduced	0.91	Reduced	Normal/HPV- at 939 days
3_32	29	ASCUS/HPV+	Negative		417	Normal/HPV-	Reduced	0.98	Reduced	
3_38	32	ASCUS/HPV+	31,33,51,62,66		404	Normal/HPV+	Reduced	1.00	Reduced	
3_39	36	ASCUS/HPV+	26,31,58,71,83		461	*Normal/HPV+	Reduced	1.00	Reduced	Normal at 872 days
4_01	43	LSIL	53,62,70,72,CP6108		495	Normal/HPV-	Reduced	1.00	Reduced	
4_02	34	LSIL	Negative		711	Normal/HPV+	Reduced	0.93	Reduced	
4_11	37	LSIL	6,7,33		511	Normal/HPV-	Reduced	0.78	Reduced	
4_13	28	LSIL	39		710	Normal	Reduced	1.00	Reduced	
4_22	43	LSIL	16,42		423	Normal/HPV-	Reduced	0.97	Reduced	
4_33	38	LSIL	39		419	Normal/HPV-	Reduced	0.91	Reduced	
5_06	29	HSIL	**Not Tested**	**LEEP**	420	*Normal/HPV-	Reduced	0.96	Reduced	Normal/HPV- at 783 days
5_15	43	HSIL	**Not Tested**	**LEEP**	457	Normal/HPV-	Reduced	0.69	Reduced	
5_27	49	HSIL	16,62	**LEEP**	655	*Normal	Reduced	1.00	Reduced	Normal at 1141 days
5_31	39	HSIL	16,68		488	*ASCUS/HPV-	Reduced	0.93	Reduced	Normal/HPV- at 908 days
5_33	36	HSIL	**Not Tested**		479	Normal	Reduced	0.99	Reduced	
5_35	28	HSIL	16,53,68,73	**LEEP**	280	*ASCUS/HPV+	Reduced	1.00	Reduced	Normal/HPV- at 637 days
3_04	32	ASCUS/HPV+	**Not Tested**		486	*LSIL/HPV+	Persistent	0.10	Persistent	ASCUS/HPV+ at 949 days
3_11	48	ASCUS/HPV+	33,53,59,62,69,71,81		502	LSIL/HPV+	Persistent	0.15	Persistent	
3_40	34	ASCUS/HPV+	62		484	LSIL/HPV-	Persistent	**0.99**	**Reduced**	
4_38	37	LSIL	51,53,56,62,66,72		516	LSIL/HPV+	Persistent	0.33	Persistent	
5_19	49	HSIL	**Not Tested**		574	HSIL	Persistent	0.37	Persistent	
5_39	34	HSIL	16,33,39,62		365	HSIL	Persistent	0.29	Persistent	

Additionally, age (column 2), Roche Linear Array HPV results (column 4), treatment received between the first and second tests (column 5), time between the two tests (column 6), the assigned label used to train the statistical classifier (column 8), the model assigned probability of transitioning to reduced risk (column 9), and the corresponding label during leave-one-out cross-validation (column 10) are shown. Results after the first follow-up are shown where available (column 11). LEEP, Loop Electrosurgical Excision Procedure; ASCUS, Abnormal Squamous Cells of Undetermined Significance; HPV+, presence of Human papillomavirus; HPV-, absence of Human papillomavirus; LSIL, -grade Squamous Intraepithelial Lesion.

**Figure 4:**
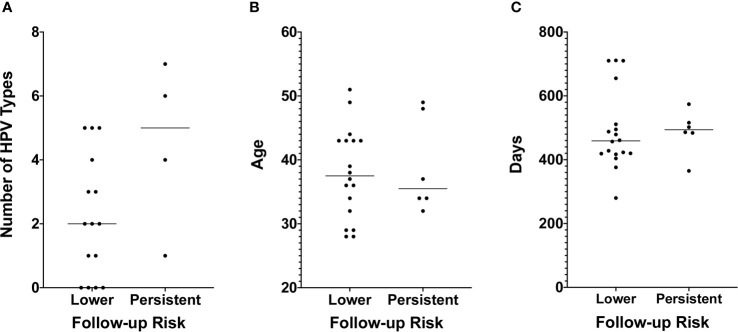
**(A)** The number of HPV types for which a sample tested positive using the Roche Linear Array. Only 19 of the 24 samples used for TCRB sequencing and for which we obtained follow-up cytology and hrHPV test results had sufficient DNA to also conduct HPV typing. The number of HPV types for which a sample tested positive is shown on the y-axis, and risk as assessed by the follow-up cytology and hrHPV results is shown on the x-axis. A test for difference in the median value between the two groups was conducted using a Mann-Whitney test. p values are reported when p ≤ 0.05. **(B)** Age is shown on the y-axis. Other details are as in Panel **(A)**. **(C)** The number of days between the first and second cytology and hrHPV test results is shown on the y-axis. Other details are as in Panel **(A)**.

While none of the six with persistent follow-up results received treatment in the period between the index and follow-up tests, only four of the 18 who transitioned to lower risk did (**Table 1**). Therefore, the different disease trajectories are not explained by differences in whether treatment was received. Fifteen of those who transitioned to lower risk and four of those who didn’t were among the 96 women for whom HPV genotyping was conducted with the Roche Linear Array (**Table 1**). We found no statistically significant difference in the median number of HPV types with which the two groups were infected (p = 0.1102 by Mann-Whitney test, **Figure 4A**), but this may be due to the small sample size, particularly for those with persistent risk. We also found no difference between the two groups with respect to the proportion of women infected with types HPV16 or HPV18, with five (30%) among those who transitioned to lower risk and one (25%) among those who did not, infected with one of the two types, and none in either group infected with both. Additionally, we found no difference in the median age or the median number of days between the first and second cytology/hrHPV tests (**Table 1**, **Figures 4B, C**).

**Figure 5:**
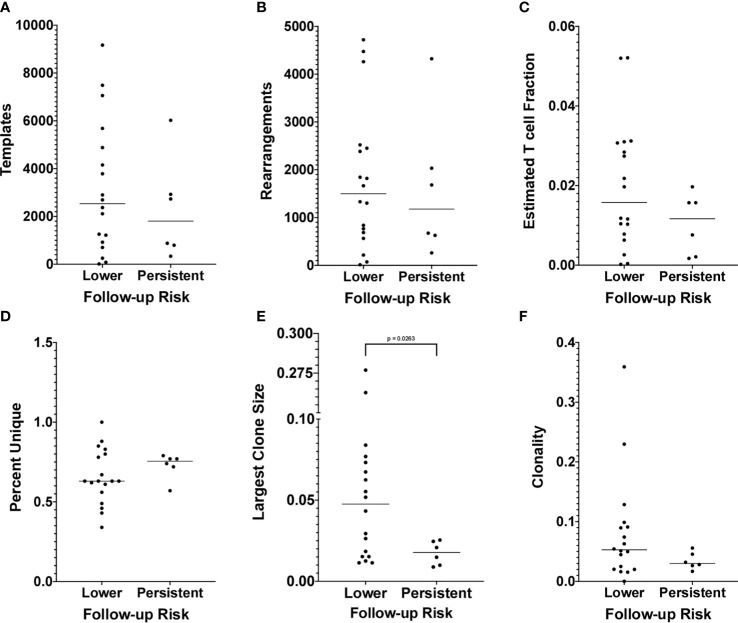
**(A)** The number of productive templates obtained for each sample is shown on the y-axis. Other details as in **Figure 4A**. **(B)** The number of productive rearrangements obtained for each sample is shown on the y-axis. Other details as in Panel **(A)**. **(C)** The Fraction Productive of Cells Mass Estimate, referred to as the Estimated T cell Fraction, obtained for each sample is shown on the y-axis. Other details as in Panel **(A)**. **(D)** The percentage of productive templates that correspond to a unique nucleotide sequence obtained for each sample is shown on the y-axis. Other details as in Panel **(A)**. **(E)** The relative abundance of the largest clone obtained for each sample is shown on the y-axis. Other details as in Panel **(A)**. **(F)** The Productive Clonality (1 – normalized Shannon Entropy) obtained for each sample is shown on the y-axis. Other details as in Panel **(A)**.

To determine whether TCR repertoire properties are associated with the transition to lower risk results, we considered the same six measures as above. We found no difference in the median values between the two groups for any measure except the size of the largest clone, for which we found that those with persistent risk had a significantly lower median largest clone size than those who transitioned to lower risk results (p = 0.0263 by Mann-Whitney test, **Figure 5**). All tests were conducted both with and without the samples with template counts below 500 with no change in results, except the p value for the largest clone size test increased to p = 0.0501 when the low-count samples were excluded. Despite the difference in median value between the two groups for this measure, there was no threshold value that would fully segregate the two groups (**Figure 5E**).

### Biophysicochemical Motifs in TCR CDR3s Predict a Transition to Lower Risk Results

**Figure 6:**
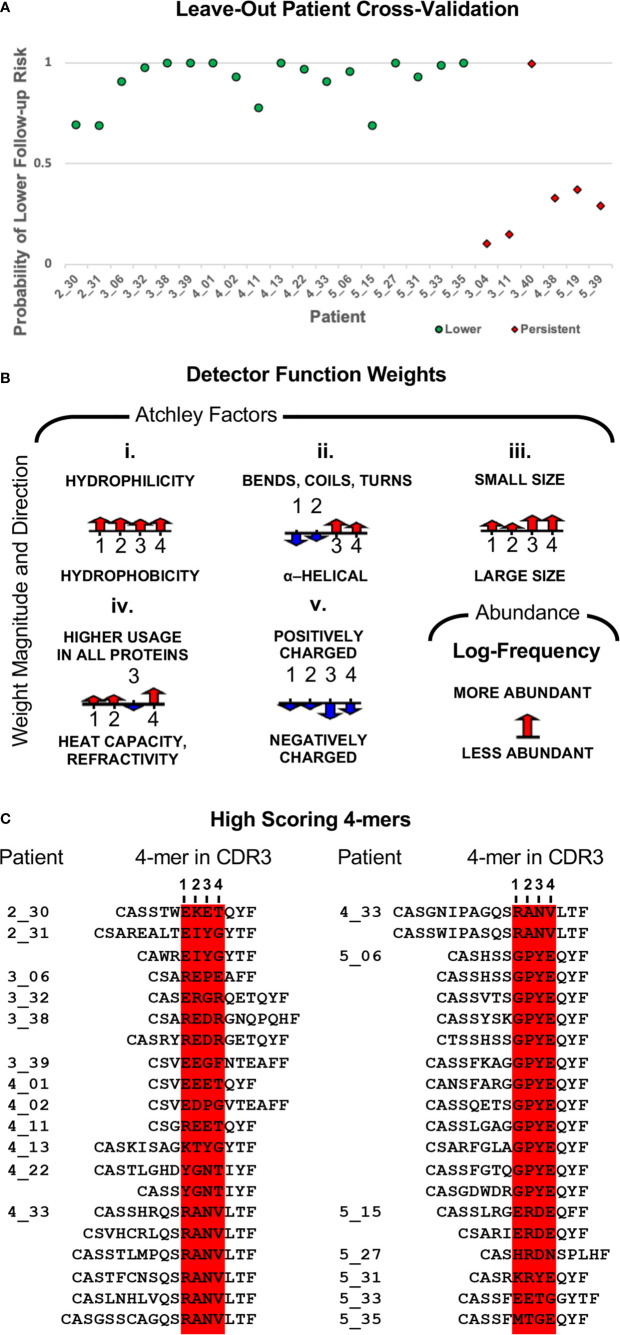
**(A)** Model classification accuracy obtained during leave-one-out cross-validation in which all sequences from one sample were held out for validation. The y-axis shows the model-assigned probability that the follow-up sample would be associated with lower risk than the index sample. The x-axis enumerates patients, and the legend indicates how the index sample was classified for model training. **(B)** Illustration of the classifier weights after fitting the model to all 24 samples. For each of the five Atchley factors, the weights are shown for the four residue positions. The weight for 4-mer relative abundance is also shown. Positive weight values are shown pointing up, and negative weight values are shown pointing down. The length of the arrow corresponds to the weight’s magnitude. **(C)** The top-scoring 4-mer for each sample is shown in the context of the CDR3s that contain it.

The above result suggests that women who transition to lower risk results have undergone more extensive clonal expansion than those with persistent risk. To determine whether the repertoires of these women include clones with shared TCR sequence patterns, we leveraged an approach we previously developed (45, 46, 50). We applied the model identified in (46), initialized weights as in (50), and used gradient optimization and cross-validation to identify a biophysicochemical motif in TCRB CDR3 sequences whose presence predicts a transition to lower risk results with 96% accuracy (23 of 24 outcomes correctly predicted) by leave-one-out cross-validation in which all sequences from one sample are held out each round (**Figures 2** and **6A**, **Table 1**). When the four women who received treatment are excluded, the accuracy is 95% (19 of 20 outcomes correctly predicted). All samples from women who transitioned to lower risk were correctly classified, as were five of six from women who did not. The single misclassified sample, 3_40, had an initial cytology and hrHPV test result of ASCUS/HPV+ and a follow-up cytology/hrHPV test result of LSIL/HPV- 484 days later. Thus, while she did not transition to normal cytology, testing for hrHPV was negative, suggesting that she may have cleared the infection or controlled the virus to below the level of detection.

To estimate the probability of correctly classifying 23 of 24 samples by chance, we performed a permutation analysis of 20 runs. For each run, the sample labels were permutated, and exhaustive leave-one-out cross-validation was performed as described above. The classification accuracies of all 20 runs were < 95%, and the average accuracy was 62%, with a range of 46% to 83% (**Supplementary Table 2**).

To determine the biophysicochemical motif properties that drive the model to assign a higher probability of transitioning to lower risk results, we examined the weights of the model when trained on all 24 samples (**Figure 6B**). The model weights reveal how each Atchley factor, each 4-mer position, and 4-mer abundance contribute to the model-assigned probability. We found that 4-mers composed of four small, hydrophilic residues result in an increased model-assigned probability of transition to reduced risk. We further found that 4-mers composed of residues that tend to form alpha-helices were preferred in the first and second 4-mer positions, while negatively charged residues that tend to form bends, coils, or turns were preferred in the third and fourth positions. Finally, we found that higher 4-mer relative abundance in a sample also increased the model-assigned probability of a transition to reduced risk.

Next, again using the model trained on all 24 samples, we identified all 4-mers in a sample scoring above the 0.5 threshold along with the CDR3s in which they are contained. Under this model, high-scoring 4-mers were only observed among the samples from women who did transition to lower risk results. There was a total of 61 high-scoring 4-mers, with the number per sample varying from one to 12, and more than half of samples having three or fewer (**Supplementary Table 3**). We note that the set of 4-mers is quite diverse. Indeed, only one 4-mer is present in more than one sample, RANV, and it is present in only two samples. There was a total of 153 CDR3s containing a high-scoring 4-mer (**Supplementary Table 4**). The number per sample ranged from one to 28, with 12 samples having eight or fewer. The top-scoring 4-mer for each sample is the 4-mer that determines the sample’s model-assigned label. Thus, we aligned the top-scoring 4-mer from each sample for which that 4-mer had a probability > 0.5 (**Figure 6C**). Each 4-mer is shown within the context of every CDR3 in which it appears. Most samples had only one or two CDR3s containing the top-scoring 4-mer, but there were two exceptions, patients 4_33 and 5_06, with six and 12 high-scoring CDR3s, respectively. We determined the relative abundance rank for each of the CDR3s and found that the most abundant CDR3 bearing a top-scoring 4-mer was among the most abundant CDR3s for every sample (**Supplementary Table 5**). For seven samples, this CDR3 was the most abundant clone in the sample, for 14 samples it was among the top 10 most abundant clones in the sample, and the lowest ranked was still among the top 25.

## Discussion

The need to improve triage of abnormal cytology and hrHPV test results during cervical cancer screening and surveillance motivated us to undertake this proof-of-principle study to determine whether T cells present in the cervical samples used for those tests could be used to refine estimates of cancer progression risk. The first goal was to demonstrate that TCR repertoires could be characterized from these samples and to provide the first such characterization. To this end, we have shown that we can obtain robust TCR repertoire data from cervical samples (**Supplementary Figure 1**). We have further shown that the commonly used repertoire-level summary statistics do not differ between the five result categories and do not differ between HPV+ and HPV- samples (**Supplementary Figures 1**–**4**). Thus, while we observed quite a bit of sample-to-sample variability in these measures, we find no evidence that there are differences between the result categories or HPV infection states in terms of lymphocyte presence or clonal expansion. We conclude that samples from all result categories are expected to be equally good candidates for the approach we are proposing.

Our second goal was to determine whether TCR repertoire or sequence properties were associated with the cytology and hrHPV test results obtained on the patient’s first follow-up sample. Thus, for the 24 of 103 women who had a second cervical sample collected following the one from which we sampled T cells, we grouped them according to whether or not the follow-up test results were of lower risk than those of the index results (**Table 1**). We found that, among the widely used repertoire-level summary statistics, there were no differences between women who transitioned to lower risk versus those of persistent risk except for a difference in the median largest clone size (**Figure 5**). We found that for women who transitioned to lower risk results, the size of the largest clone in the sample was much larger on average than for women who did not, suggesting that their T cell repertoires had undergone more extensive clonal expansion for at least one clone in the local cervical T cell response. While finding a difference in the median largest clone size may seem to contradict the lack of a difference in median clonality, we suspect this is due to the fact that clonality is a repertoire-level measure that attempts to quantify the extent to which the distribution of all clonal relative abundances in a repertoire departs from a uniform distribution. That is a different question than asking whether the median size of the largest clone is different between two groups of repertoires. We did, however, find that, while the median clonality was not statistically significantly different between the two groups, there is a trend toward higher clonality for the group of women who transitioned to lower risk results (**Figure 5F**). Both the larger median largest clone size and the trend towards higher clonality could be indicative of a more robust or targeted antigen-driven immune response. Despite this difference, however, there is no largest clone size that completely separates the two groups of women, as the lower range of values for women who transitioned to lower risk results overlaps the full range of values for women who did not. This is analogous to the result by Cui et al. (52) who found that the average number of unique amino acid TCR sequences in sentinel lymph nodes differed between women with cervical cancer who experienced disease progression versus though who did not, but despite this difference, there was significant overlap in the ranges of values between the two groups of women making it difficult to gain any predictive capability from the measure.

Based on the above results, we concluded that, while a high largest clone size indicates a high likelihood of transitioning to a lower risk combination of cytology and hrHPV test results, a low largest clone size appears to be equally associated with both lower and persistent risk at follow up. Thus, to increase the accuracy of identifying women likely to persist, we built on our prior work developing statistical classifiers that accurately classify TCR repertoires according to sequence-level properties present within the repertoire (45–47, 50). The basis of our method is identification of a biophysicochemical motif in TCR CDR3 sequences that is shared across samples with a common phenotype. In this study, we identified a motif whose presence in TCRB CDR3 sequences marked cervical repertoires collected from women who later transitioned to lower risk cytology/hrHPV result categories. Similarly, the absence of this motif marked cervical repertoires collected from women whose risk level persisted. The motif served as a biomarker of future lower risk with 96% accuracy (23/24) by exhaustive leave-one-out cross-validation in which all sequences from a sample are held out each round (**Figure 6A**). The accuracy calculated over the 20 women who did not receive treatment (**Table 1**) is 95% (19/20). The average classification accuracy over 20 permutation runs was 62%, and all runs had an accuracy < 95% (**Supplementary Table 2**). Thus, we conclude that the probability of observing a cross-validation classification accuracy of 95% or higher by chance is p < 0.05.

Both the 96% and the 95% accuracies are a result of misclassifying a single sample, 3_40. This sample was misclassified according to the labels we assigned and used for training the model. We classified this woman as having persistent or increased risk, because the index cytology result was ASCUS and the follow-up cytology result was LSIL (**Table 1**). In contrast, the model assigned a probability of 0.99 that this woman would experience a transition to lower risk, and indeed she transitioned from hrHPV+ to hrHPV- (**Table 1**). Thus, she may indeed be transitioning to lower risk cytology/hrHPV result combinations.

In the model, we represented amino acid 4-mers from TCRB CDR3 sequences according to their biophysicochemical properties using Atchley factors in order to capture properties of the CDR3 sequence that determine the CDR3’s peptide-binding specificity, as motivated in (46). The shared motif is described by the model weights indicating the relative importance of the different amino acid properties, different 4-mer positions, and the 4-mer’s abundance in the sample to the calculated probability that the 4-mer is derived from a TCRB sequence found in a repertoire sampled from a woman who transitioned to lower risk (**Figure 6B**). We found that 4-mers composed of four small, hydrophilic residues result in an increased probability of a reduced risk classification. The probability of a reduced risk classification is further increased for 4-mers composed of residues that tend to form alpha-helices in the first and second 4-mer positions and positively charged residues that tend to form bends, coils, or turns in the third and fourth positions. Finally, we found that higher 4-mer relative abundance in a sample also increased the probability of a reduced risk classification. The properties of this motif are distinct from the three other TCR motifs we have identified with this method, and indeed all four motifs are distinct (46, 50).

We hypothesize that the motifs we have identified mark TCRBs that have undergone clonal expansion as part of an antigen-specific immune response critical to the resolution of cervical lesions and/or clearance of hrHPV infection. This hypothesis is supported by the finding that in all women with high-scoring motifs, the clone bearing the woman’s top-scoring 4-mer/motif is one of the most abundant clones in the sample (**Supplementary Table 5**). Indeed, in many cases, it is the most abundant. The most obvious hypothesis is that the relevant antigen is an HPV antigen and the corresponding immune response critical to clearing infection. An intriguing alternative is that the motif is associated with the presence of particular microbes in the cervical microbiome, such as *Lactobacillus iners*, which has been associated with the clearance of hrHPV, or *Gardnerella*, which has been associated with progression (66). Finally, there could be antigens expressed on cells with abnormal cytology and the corresponding immune response critical to eliminating these abnormal cells. A comparison of the motif-bearing CDR3 sequences (**Figure 6C**) against databases of curated TCR sequences with known specificities revealed no matches (67–69). Thus, experimental studies will be required to determine whether these motifs mark a TCR specificity relevant to the process of lesion resolution or infection clearance, and, if so, what that antigen is.

While the motifs we have identified are associated with a transition to lower risk, both Lang Kuhs et al. (51) and Cui et al. (52) identified sequences associated with CIN or cancer. Lang Kuhs et al. observed that the TCRBV6-7 gene segment was overrepresented among women with HPV16-associated CIN3 (51). Thus, we compared the TCRBV6-7 relative abundance between cervical cytology samples with a normal versus HSIL reference diagnosis, between women who transitioned to lower risk and women whose risk level remained unchanged, and between women who were HPV16+ versus HPV16-. In all cases, we found no difference. We further found that none of our motif bearing CDR3s (**Supplementary Table 4**) were part of rearrangements that included TCRBV6-7. Key differences between our study and the Lang Kuhs et al. study that could explain the different results is that our study included women infected with a variety of HPV types and our repertoires were derived from cytology rather than blood samples. Cui et al. (52) found CDR3 sequences that were shared exclusively among patients with CIN or exclusively among patients with cervical cancer, with sharing ranging from 9-11 and 8-9 patients, respectively. None of these CDR3 sequences were found in any of our sampled repertoires. This is not necessarily surprising. Cui et al. had sample sizes of 30 CIN patients and 25 cancer patients. Thus, even the most broadly shared CDR3 sequences were not observed in more than half of the patients. Furthermore, our failure to observe these sequences may be a consequence of sample the cervical rather than peripheral blood repertoire.

There are multiple factors with the potential to confound our results, differences between the two groups of women with respect to race/ethnicity, age, HPV infection, time between the two sets of results used to determine disease trajectory, and whether treatment was received between them. We have controlled for or assessed the potential impact of each of these as much as possible. First, our patient cohort consists entirely of White Hispanic women. This not only controls for the known confounding factor of race/ethnicity in cervical cancer risk but also provides a valuable data resource for future studies aimed at elucidating the causes of cervical cancer disparities. Regarding age, while we find that women with an LSIL diagnosis for the reference cytology result have a significantly lower median age than women with a normal reference cytology result (**Figure 3A**), we found no difference in the median age between women who transitioned to lower risk on follow-up and those who did not (**Figure 4B**). We found no difference between the two groups of women for the amount of time between the first and follow-up cervical cytology/hrHPV results, or the number of HPV types with which an individual was infected (**Table 1**, **Figure 4**). We further found no difference in HPV16 or HPV18 positivity between the two groups (**Table 1**). Finally, receipt of excisional treatment for precancer also does not appear to explain the difference, given that only four of 24 patients received treatment between the two sets of results (**Table 1**). Among the 20 untreated women, there are 14 in the reduced risk category and 6 in the persistent risk category, and the leave-one-out cross-validation accuracy excluding the four treated patients is 95% (19/20). Overall, our sample, while small, has similar representation for each of these factors in both groups, and/or the groups themselves do not differ on the basis of these factors. Thus, we conclude that other factors are contributing to the difference in apparent disease trajectory between the two groups sampled in this study. Additional potential confounders that we were unable to account for include HLA type, smoking status, and a history of HPV vaccination. These need to be accounted for in a future study with a larger sample size.

All four of the women who received treatment between their reference and follow-up cytology/hrHPV results received lower risk results on follow-up. They were also predicted to have lower follow-up risk by our model. It is important to note, however, that their transition to lower risk may be driven solely by the treatment and may not reflect the biological state of their lesion, HPV infection, or T cell response at the time of sample collection. Only ~9% of women receiving excisional treatment show residual or recurrent abnormal cytology or persistent hrHPV infection after treatment, compared to 40% to 80% of untreated women, depending on age and lesion grade (70).

The above-presented evidence from the 20 untreated women is consistent with our original hypothesis that women who experience spontaneous regression of cervical lesions and clearance of HPV infection do so, at least in part, as a consequence of an antigen-specific, T cell-mediated immune response that can be detected *via* TCR sequence patterns. There are limitations of our study, however, the primary ones being the small training set size and the lack of a test set. We fully expect the result here to hold, however, given that our prior results with similarly sized training sets have shown model generalizability on test data (45, 50). Indeed, the T cell study (50) had 95% accuracy by cross-validation and 80% accuracy on an independent test set. More importantly, we note that there was no model selection in the current study. Here, we used the model selected in (46) and only used the training set to fit the model parameters (**Figure 2**). Thus, we expect test set accuracy greater than 80%. Additionally, we expect much higher accuracy with a large training set (71). Indeed, the purpose of this proof-of-principle study was precisely to determine whether a large-scale study is justified, and the results presented here do indeed warrant follow up in a large study. Unfortunately, given the longitudinal nature of our study design and the relative rarity of disease trajectories of persistent risk, such a study is expected to take many years.

One potential limitation of our study is the use of cytology results without histologic confirmation. We used cytology rather than histology results, because, per guidelines, histologic diagnosis is only recommended for a subset of abnormal results. Thus, in this proof-of-principle study using standard-of-care data, cytology results (plus reflex hrHPV test results) are the only result types consistently available for all women across all abnormal and normal result combinations. Indeed, there would be ethical questions around designing a research study in which histologic confirmation was obtained for all results, even just for all abnormal results. We do, however, provide histologic diagnoses as well as repeat follow-up cytology and hrHPV test results where those are available (**Supplementary Table 6**). Furthermore, risk-based management relies heavily on cytology and hrHPV test results for clinical decision making. Thus, our goal is to maximize the amount of information about risk that can be obtained from cervical cytology samples.

Additional limitations of our study include the use of cytology samples that had been stored at room temperature in PreservCyt solution for five weeks before processing, the focus on a single race/ethnicity, and the focus on only a single TCR chain (TCRB). We recommend that future studies building on the results presented here utilize freshly collected research samples that would be expected to have better DNA yield as well as to permit RNA-based studies. RNA-based studies could utilize single-cell RNA sequencing which would enable joint analysis of a T cell’s gene expression profile (including T cell type and subset) with its full TCR (both α and β chains). Additionally, replication of this finding across multiple races and ethnicities is needed. The results of this proof-of-principle study provide justification for pursing these aims.

Determination of whether a similar signal can be detected in blood would inform whether development of a blood-based assay would be possible. In our view, given the benefits of conducting all tests on a single sample, pursuing application of this approach to blood would only make sense if it was determined that cervical cytology sampling was no longer needed. We would not expect a blood-only approach to be fruitful given the increased repertoire diversity in blood and the consequent rarity of cervix-associated T cells in blood. This is supported by current evidence indicating that HPV16 reactivity among peripheral blood T cells is not associated with regression in women with HPV16 CIN 2/3 (53). The Trimble et al. (53) study should not be taken as definitive, however, due to the limited follow-up period and the fact that only HPV16 E6/E7 antigens were tested. Other antigens may be relevant, as discussed above. Furthermore, after discovering a signal in the cervical repertoire, optimized approaches for detecting it in blood can be designed. Thus, a study to determine the amount of overlap between the cervical and blood T cell repertoires may be warranted.

The results presented here are significant both because they represent a significant advance in the field of immune repertoire predictive modeling as the first to demonstrate the potential to predict a future outcome, but also because they have the potential to stimulate increased interest in coupling the current focus on lesion formation (cytology testing) and persistent hrHPV infection (hrHPV testing) with factors mediating regression, i.e., a T cell-mediated antigen-specific immune response. Given the high proportion of cases that undergo regression, including an assay that triages samples based on the potential for future regression rather than focusing solely on the current presence of a lesion or its causal agent could significantly improve screening effectiveness with significant benefits both for populations that currently receive guideline-recommended screening as well as for under-screened populations. In screened populations (i.e., in high resource settings), testing is recommended every three to five years unless an abnormal result is obtained, at which point there is a shift to more frequent surveillance testing and a recommendation for treatment of women with a CIN3 risk of 26% or higher (10–12). While this strategy has significantly reduced cancer incidence and mortality in screened populations, it is accompanied by overtreatment in the form of unnecessary procedures that have their own risks and complications, particularly among women of reproductive age. Thus, the ability to reduce the frequency of surveillance screening and the number of unnecessary treatments is expected to reduce this overtreatment burden on screened women (27–31). More importantly, the current source of cervical cancer resides primarily in unscreened or under-screened populations, and here an approach that triages samples based on the potential for future regression has the potential for even greater impact. To deliver effective screening to under-screened populations, there has been a focus on devising strategies for preventing the most cancers *via* a single screening encounter (23–26). The current strategy focuses on deploying a one-time test at an age where the test is most likely to catch persistent infections and pre-cancers, such as between the ages of 30 and 39 years. This one-time strategy recognizes that it would not prevent cancers occurring before the screening age. The ability to distinguish women likely to regress from those at highest risk of progressing to cancer might allow the one-time test to be deployed at a younger age, thereby preventing a larger number of cancers. Additionally, it could facilitate decision making for screen-positive women allowing focused follow-up on those most at risk of progression. Thus, while the effectiveness of one-time testing remains an open question, the development of assays to help identify women likely to regress is expected improve delivery of screening to under-screened populations by increasing the amount of information about a woman’s risk that is obtained at each screening encounter. Deployment of such an assay would only be feasible if it could be designed for use in low resource settings. However, if this could be accomplished, it would be expected to contribute towards a reduction in cervical cancer incidence and mortality.

## Data Availability Statement

The TCRB sequencing data are freely available from the VDJServer Community Data Portal (CDP) (http://vdjserver.org) under the project accession 4995411523885404651-242ac118-0001-012 (56). The study metadata are consistent with the AIRR C MiAIRR Standard (72). VDJServer is part of the AIRR C Data Commons (73).

## Ethics Statement

The studies involving human participants were reviewed and approved by Institutional Review Board of the University of Texas Southwestern Medical Center. Written informed consent for participation was not required for this study in accordance with the national legislation and the institutional requirements.

## Author Contributions

SC and LC wrote the manuscript. SC, JO, WZ, and BS performed experiments. SC, JT, EL and LC analyzed and interpreted the patient data. LQ handled all IRB document processing and sample retrieval. NM, AG, JT, EL, and LC designed the work. All authors contributed to the article and approved the submitted version.

## Funding

This research was supported by Simmons Comprehensive Cancer Center Development Funds and by a charitable donation from Young Texans Against Cancer, both to LC and JT.

## Conflict of Interest

The authors declare that the research was conducted in the absence of any commercial or financial relationships that could be construed as a potential conflict of interest.

## Supplementary Material

Supplementary Figure 1
**(A)** The number of productive templates obtained for each sample. Number is shown on the y-axis, and result category is shown on the x-axis. The horizontal dotted line indicates a template count of 5000. Other details are as described for **Figure 3A**. **(B)** The number of productive rearrangements obtained for each sample. Other details as in Panel **(A)**. **(C)** The Fraction Productive of Cells Mass Estimate, referred to as the Estimated T cell Fraction, obtained for each sample. Other details as in Panel A.

Supplementary Figure 2
**(A)** The number of productive templates obtained for each sample. Number is shown on the y-axis, and HPV infection status according to the Roche Linear array is shown on the x-axis. Samples are classified as positive if they are positive for one or more HPV types. A test for difference in the median value between the two groups was conducted using a Mann-Whitney test. p values are reported when p ≤ 0.05. **(B)** The number of productive rearrangements obtained for each sample. Other details as in Panel **(A)**. **(C)** The Fraction Productive of Cells Mass Estimate, referred to as the Estimated T cell Fraction, obtained for each sample. Other details as in Panel **(A)**.

Supplementary Figure 3
**(A)** The percentage of productive templates that correspond to a unique nucleotide sequence (Percent Unique) obtained for each sample. Percentage is shown on the y-axis, and result category is shown on the x-axis. Other details are as described for **Figure 3A**. **(B)** The relative abundance of the largest clone (Largest Clone Size) obtained for each sample. Other details as in Panel **(A)**. **(C)** The Productive Clonality (1 – normalized Shannon Entropy) obtained for each sample. Other details as in Panel A.

Supplementary Figure 4
**(A)** The percentage of productive templates that correspond to a unique nucleotide sequence (Percent Unique) obtained for each sample. Percentage is shown on the y-axis, and HPV infection status according to the Roche Linear array is shown on the x-axis. Other details are as described for **Supplementary Figure 2A**. **(B)** The relative abundance of the largest clone (Largest Clone Size) obtained for each sample. Other details as in Panel **(A)**. **(C)** The Productive Clonality (1 – normalized Shannon Entropy) obtained for each sample. Other details as in Panel **(A)**.
