# Supplementary figures and images for: T Cell Receptor Repertoires Acquired via Routine Pap Testing May Help Refine Cervical Cancer and Precancer Risk Estimates
Source: Front Immunol. 2021 Apr 2;12:624230. doi: 10.3389/fimmu.2021.624230 (PMC8050337; doi:10.3389/fimmu.2021.624230)

Supplementary Figure 1

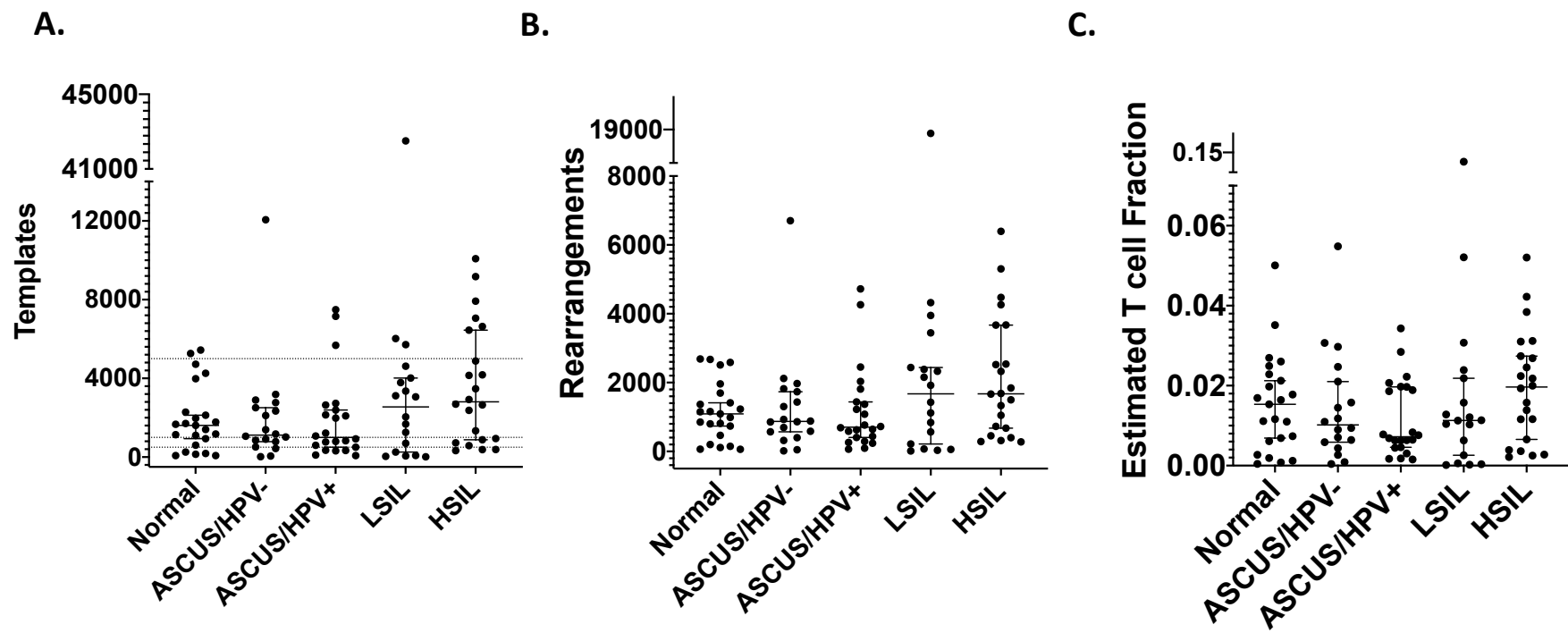

Supplementary Figure 2

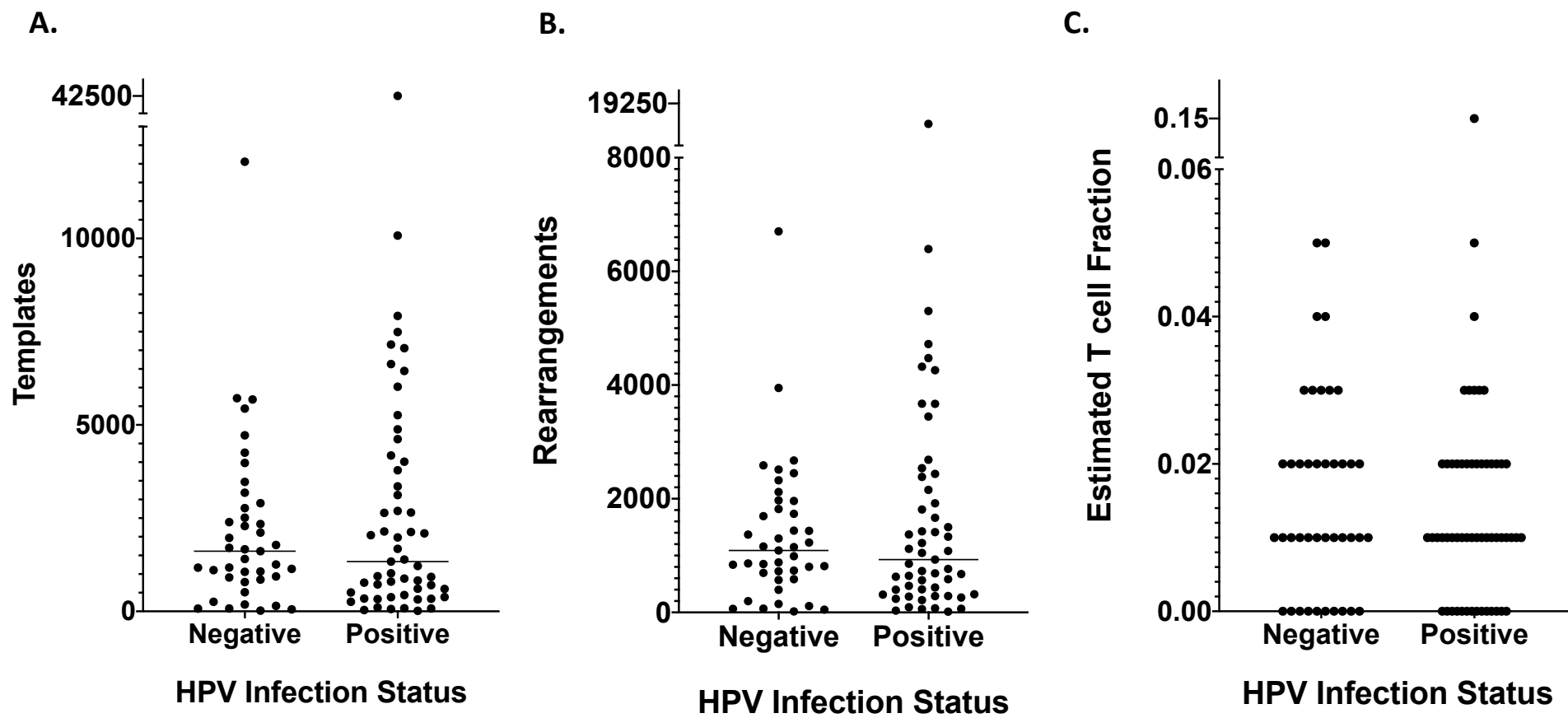

Supplementary Figure 3

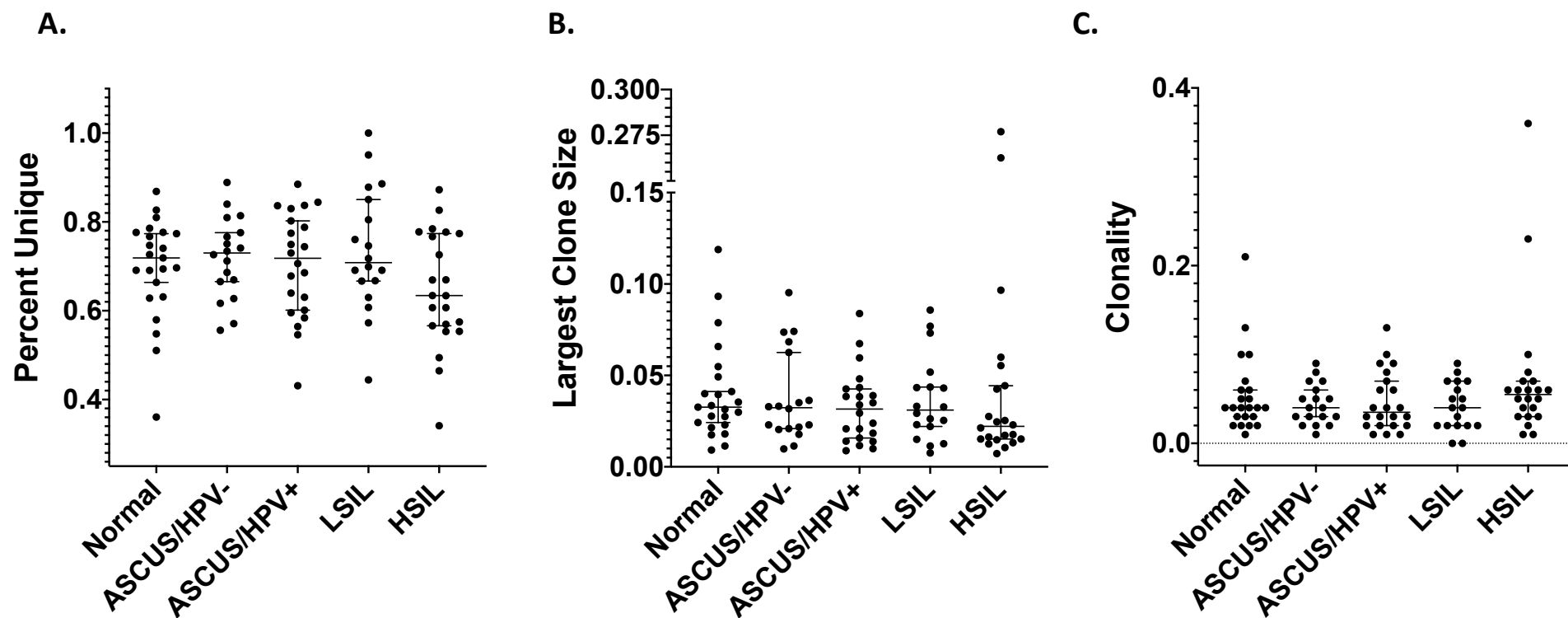

Supplementary Figure 4

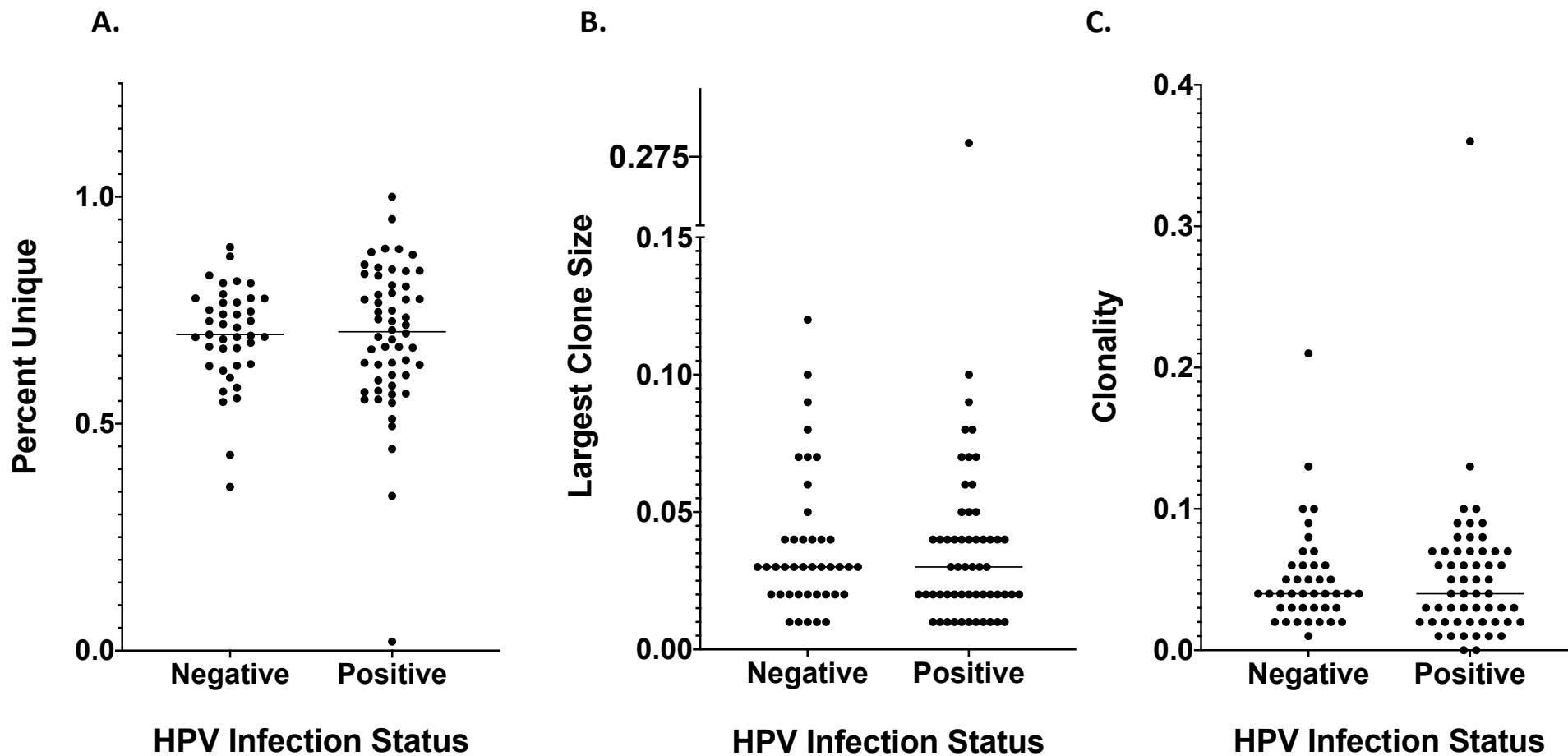

Supplement: Supplementary Figure 1 — (A) The number of productive templates obtained for each sample. Number is shown on the y-axis, and result category is shown on the x-axis. The horizontal dotted line indicates a template count of 5000. Other details are as described for Figure 3A . (B) The number of productive rearrangements obtained for each sample. Other details as in Panel (A). (C) The Fraction Productive of Cells Mass Estimate, referred to as the Estimated T cell Fraction, obtained for each sample. Other details as in Panel A. [file DataSheet_1.pdf]
